# Impact of Co-Occurring Psychiatric Comorbidities and Substance Use Disorders on Outcomes in Adolescents and Young Adults with Opioid Use Disorder: A Retrospective Cohort Study

**DOI:** 10.3390/ph18050609

**Published:** 2025-04-23

**Authors:** Ligang Liu, Erin R. McKnight, Andrea E. Bonny, Heqing Tao, Pujing Zhao, Milap C. Nahata

**Affiliations:** 1 Institute of Therapeutic Innovations and Outcomes (ITIO), College of Pharmacy, The Ohio State University, Columbus, OH 43210, USA; liu.10645@osu.edu (L.L.); zhao.4556@osu.edu (P.Z.); 2 Division of Adolescent Medicine, Nationwide Children’s Hospital, Columbus, OH 43210, USA; erin.mcknight@nationwidechildrens.org (E.R.M.); andrea.bonny@nationwidechildrens.org (A.E.B.); 3 Department of Pediatrics, College of Medicine, The Ohio State University, Columbus, OH 43210, USA; 4 Department of Gastroenterology, The First Affiliated Hospital of Guangzhou Medical University, Guangzhou Medical University, Guangzhou 510182, China; tao_heqing@163.com; 5 Departments of Internal Medicine and Pediatrics, College of Medicine, The Ohio State University, Columbus, OH 43210, USA

**Keywords:** opioid use disorder, adolescents and young adults, mental health disorders, substance use disorders, retention time, urine drug tests, medication-assisted treatment

## Abstract

**Background/Objectives:** Adolescents and young adults (AYAs) with opioid use disorder (OUD) frequently have co-occurring psychiatric conditions and substance use disorders (SUDs). This study evaluated the association of psychiatric comorbidities and other SUDs with treatment retention and urine drug test (UDT) results in AYAs with OUD. **Methods:** This retrospective cohort study included AYAs enrolled in the Substance Use Treatment and Recovery clinic from 2009 to 2022. Participants were categorized into four groups: no comorbidities, only mental health disorders, only other SUDs, and both disorders. Treatment outcomes included retention time and UDT results for medication for OUD (MOUD) and illicit substances, including tetrahydrocannabinol (THC). Kruskal–Wallis tests were used to evaluate differences across groups, and regression models identified variables associated with outcomes. Statistical significance was set at *p* < 0.05. **Results:** Among 157 patients, the median retention time was 300 days. Depression (*p* = 0.04), post-traumatic stress disorder (*p* = 0.002), and alcohol use disorder (*p* = 0.04) were associated with prolonged retention, whereas cannabis use disorder predicted shorter retention (*p* = 0.02). The median proportion of positive UDTs was 0.9 for MOUD, 0.1 for illicit substances, and 0.0 for THC. Older age (*p* = 0.02) and the use of antidepressants and anxiolytics were associated with greater adherence to MOUD. Cannabis use disorder (*p* = 0.02) and male sex (*p* = 0.04) predicted positive UDTs for THC, while MOUD use was linked to lower THC positivity (*p* = 0.02). The main limitations of this study were related to its retrospective study design and single-center setting. **Conclusions:** Psychiatric and substance use comorbidities significantly influence retention and treatment adherence in AYAs with OUD. Integrated treatment may improve engagement and outcomes. Further research is needed to tailor interventions for AYAs with co-occurring disorders.

## 1. Introduction

Adolescents and young adults (AYAs) are affected by opioid use disorder (OUD). In 2019, about 7.6 million adolescents and adults were diagnosed with OUD in the US [1]. Overdose death rates among AYAs increased by 49% from 2019 to 2020 [2]. OUD leads to a substantial economic burden as it affects not only AYAs but also their families and communities [3]. Opioid misuse and abuse in AYAs is especially concerning because it might impair cognitive function and increase the risk of future substance misuse [4].

Studies have shown that more than 50% of AYAs with substance use disorders (SUDs) experienced at least one co-occurring mental health disorder [5]. Among individuals with OUD, mood and anxiety disorders were the most prevalent mental health disorders, and benzodiazepine, cannabis, cocaine, and amphetamine were commonly used substances [6,7]. Treatment of OUD in AYAs emphasizes integrated mental health and addiction care across treatment settings [5]. The goal of integrated treatment is to provide continuous, individualized care that emphasizes recovery principles and addresses the unique needs of each patient [8]. This approach includes the use of pharmacological treatments with psychiatric interventions, leading to enhanced patient health outcomes [9]. In addition, all AYAs with OUD should be offered evidence-based medication treatment for OUD (MOUD) as part of a comprehensive and integrated treatment approach [10].

Retention time is an essential component of successful OUD treatment, as longer retention has been associated with significantly improved outcomes, including reduced morbidity and mortality [11]. Urine drug test (UDT) is a key monitoring tool in OUD management. Periodic UDT results have provided objective data to monitor adherence to MOUD, identify changes in drug use patterns, and detect early relapses [12].

Conflicting results have been reported regarding the impact of psychiatric disorders on retention time, medication adherence, and relapse among patients with OUD [13,14,15,16,17]. Some studies indicated that patients diagnosed with psychiatric disorders exhibited significantly lower adherence to MOUD and higher relapse rates compared to those without psychiatric disorders [13,17]. On the other hand, other research found that individuals with co-occurring psychiatric disorders had greater improvements in abstinence [14,15,16]. Moreover, most existing studies have focused primarily on adult populations, not AYAs. Given these gaps and conflicting results, this study aimed to evaluate the impact of co-occurring psychiatric comorbidities and other SUDs on retention time, positive UDT for MOUD, and positive UDT for illicit substances in AYAs with OUD.

## 2. Results

The study included 157 AYAs with OUD who were categorized into four groups: no comorbidities (N = 26), mental health disorders only (N = 45), other SUDs only (N = 37), and both mental health disorders and SUDs (N = 49). The mean age of participants was 18.5 ± 1.9 years. Most patients were White (94.9%) and female (60.5%). Medicaid/sCHIP was the most common type of insurance (45.2%). Anxiety (38.2%), depression (33.1%), ADHD (14.6%), bipolar disorder (5.1%), and PTSD (4.5%) were the most prevalent mental health disorders. Medications were commonly prescribed for these conditions, with 87.5% receiving medication for bipolar disorder, 80.8% for depression, 78.3% for anxiety, and 59.1% for ADHD. Regarding SUDs, 7.6% of patients were diagnosed with nicotine use disorder, 12.1% with cannabis use disorder, and 43.3% with other SUDs. Most patients (88.5%) received buprenorphine-naloxone for OUD, while 9.6% were treated with a combination of buprenorphine-naloxone and naltrexone (not simultaneously). Only 1.3% of patients did not receive any MOUD. Demographic and clinical characteristics of the study population are presented in Table 1.

**Figure 1:**
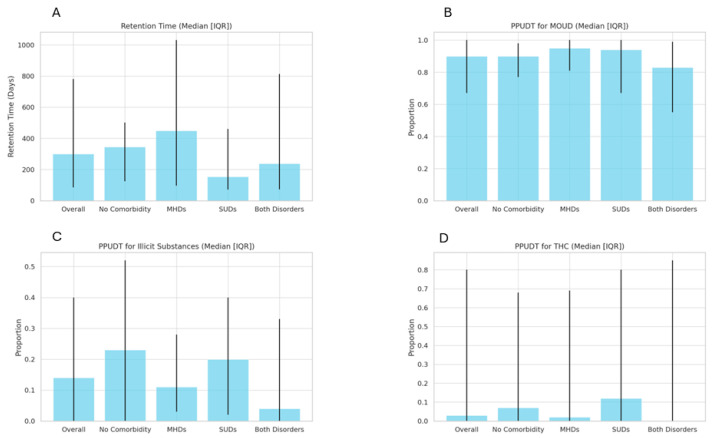
(**A**): Retention time across patient groups; (**B**): proportion of positive urine drug tests (PPUDT) for MOUD Across Patient Groups; (**C**): proportion of positive urine drug tests (PPUDT) for illicit substances across patient groups; (**D**): proportion of positive urine drug tests (PPUDT) for THC across patient groups. Abbreviations: MHDs: mental health disorders; SUDs: substance use disorders; UDTs: urine drug tests; PPUDT: proportion of positive urine drug tests; MOUD: medication for opioid use disorder; THC: tetrahydrocannabinol.

The overall median retention time was 300 days (IQR: 85, 781). Patients without comorbidities had a retention time of 345 days (IQR: 124, 501), patients with only mental health disorders had the longest retention time of 449 days (IQR: 97, 1031), those with only SUDs had a retention time of 154 days (IQR: 71, 460), and those with both mental health disorders and SUDs had a retention time of 238 days (IQR: 73, 813). There were no statistically significant differences in retention time across the groups (*p* = 0.56) (Figure 1A).

We examined sex differences in retention time and urine drug test outcomes. There were no significant differences for the mean retention time, median proportion of positive UDT for MOUD, or median proportion of positive UDT for illicit substances (*p* = 0.138, *p* = 0.378 and *p* = 0.216, respectively). However, the median proportion of positive UDT for THC was significantly higher among males compared to females (*p* = 0.024). 

Patients with depression had a significantly longer retention time compared to those without depression (coefficient = 220.3; 95% CI, 6.8 to 433.8; *p* = 0.04). Similarly, a diagnosis of PTSD was associated with extended retention time (coefficient = 758.3; 95% CI, 279.8 to 1236.9; *p* = 0.002). Alcohol use disorder was associated with a significantly longer retention time (coefficient = 787.6; 95% CI, 54.5 to 1520.8; *p* = 0.04), whereas cannabis use disorder was associated with a shorter retention time (coefficient = −357.5; 95% CI, −664.5 to −50.5; *p* = 0.02). Treatment of anxiety with anxiety medications was strongly associated with longer retention (coefficient = 573.1; 95% CI, 179.1 to 967.1; *p* = 0.006), as shown in Table 2. 

The overall median proportion of positive UDT for MOUD was 0.9 (IQR 0.67, 1.0). The medians were 0.9 (IQR 0.77, 0.98) for patients without comorbidities, 0.95 (IQR 0.81, 1.0) for those with mental health disorders, 0.94 (IQR 0.64, 1.0) for those with other SUDs, and 0.83 (IQR 0.55, 0.99) for those with both mental health disorders and SUDs. The differences were not statistically significant between the four groups (*p* = 0.25). The overall median proportion of positive UDT for illicit substances was 0.14 [IQR 0.0, 0.4], with no significant group differences (*p* = 0.33). The overall median proportion of positive UDT for THC was 0.0 (IQR 0.0, 0.8), with no significant differences between groups (*p* = 0.37) (Figure 1B–D).

Older age was a significant predictor of positive UDT for MOUD (coefficient = 0.16; 95% CI, 0.02 to 0.29; *p* = 0.02). Taking medications for depression (coefficient = 0.28; 95% CI, 0.09 to 0.48; *p* = 0.006) and anxiety (coefficient = 0.40; 95% CI, 0.24 to 0.57; *p* < 0.001) were also strong predictors of positive UDT for MOUD. No significant factors were identified for predicting positive UDT for illicit drugs other than THC. Male sex was significantly associated with an increased likelihood of testing positive for THC (coefficient = 0.14; 95% CI, 0.01 to 0.27; *p* = 0.04). Cannabis use disorder was a strong predictor of positive THC results (coefficient = 0.23; 95% CI, 0.03 to 0.42; *p* = 0.02). Treatment with buprenorphine-naloxone (coefficient = −0.69; 95% CI, −1.26 to −0.12; *p* = 0.02) and buprenorphine-naloxone and naltrexone (coefficient = −0.62; 95% CI, −1.22 to −0.02; *p* = 0.04) were associated with significantly lower odds of positive UDT for THC, as detailed in Table 2.

## 3. Discussion

A critical finding in this study was the longer retention observed in AYAs with co-occurring mental health disorders, particularly depression and PTSD. Our findings were consistent with prior literature among adults with OUD. Previous reports demonstrated that co-occurring PTSD was associated with higher adherence to MOUD among African American adults enrolled in substance use treatment programs [18]. Managing PTSD and OUD simultaneously may enhance patient outcomes significantly [19]. Similarly, adults with depression had longer retention in the clinic compared to those without depression [20]. This positive influence of depression on patient engagement in treatment for OUD might have occurred due to buprenorphine’s potential antidepressant properties, which could help alleviate depression and support in overcoming opioid dependence [21].

We observed that most of the participants in our clinic were females. While epidemiologic data generally showed higher rates of substance use disorders among males, treatment-seeking behaviors were more likely to occur among females [22]. This may reflect a higher likelihood among young females to seek treatment compared to males, as the previous literature suggests females are more likely to access healthcare services for substance use disorders [23]. Males with OUD are often more likely to use multiple substances [24]. In contrast, females are more likely to experience co-occurring mental health disorders [25]. Therefore, treatment programs should consider incorporating tailored behavioral interventions, integrating gender-specific motivational interviewing techniques and counseling to address underlying factors that contribute to continued substance use. Understanding how males and females respond differently to psychiatric interventions and MOUD is important to develop personalized treatment strategies for AYAs with OUD.

We did not find an association between anxiety and retention time; however, taking medication for anxiety was associated with increased retention time, suggesting that treatment of anxiety is beneficial. Our findings are consistent with a previous study, which found no association between anxiety and treatment retention [26]. Anxiety often exacerbates opioid misuse by heightening distress responses, and sensitivity to anxiety can predict treatment dropout [27,28]. Evidence indicated that addressing anxiety-specific symptoms could reduce dropout rates and improve treatment outcomes [29]. This suggests that appropriate pharmacologic management of anxiety may mitigate some challenges associated with anxiety in OUD treatment.

This study revealed that co-occurring alcohol use disorder was associated with higher retention time. However, previous research found co-occurring alcohol dependence was associated with a lower six-month treatment retention rate compared to patients aged 12–64 years without alcohol use disorder [30]. This discrepancy may be attributed to variations in age and the severity of these disorders among patients. Conversely, we found that cannabis use disorder was linked to lower retention time. Other studies also observed that baseline cannabis use was significantly linked to increased dropout rates in treatment programs for adults, particularly in heavy users [31]. Cannabis use can impair cognition and alter judgment [32], which can subsequently lead to worse treatment outcomes.

This study also found consistently positive UDTs for MOUD across most groups, with no significant differences between those with or without comorbidities. This indicated that patients were adherent to MOUD regimens, regardless of existing comorbidities. Our findings showed that older age was associated with improved adherence to MOUD. Older adolescents and young adults may possess more developed self-management skills, contributing to better treatment adherence [33]. This aligned with previous research demonstrating a positive association between age and retention in medication-assisted treatment for OUD [34,35]. Additionally, we observed that taking medications for depression and anxiety can improve medication adherence. Antidepressant use has improved treatment adherence and outcomes for adults with co-occurring depression and substance use disorders [36]. A meta-analysis concluded that antidepressants were beneficial for adults with both depression and SUDs [37]. Furthermore, nonadherence to psychiatric medications was associated with shorter treatment stays and poorer outcomes in adults with OUD [38]. 

The overall low median proportion of positive UDTs for illicit substances suggests that 90% of patients were abstaining from illicit substances during treatment. However, we found that male sex and cannabis use disorder were associated with higher odds of testing positive for THC. Males have had higher rates and frequency of cannabis use [39]. More importantly, AYAs receiving buprenorphine-naloxone or its combination with naltrexone had lower odds of testing positive for THC, which aligned with previous studies showing a decline in cannabis use during opioid maintenance therapy in adults [40,41,42]. This suggests that MOUD may provide ancillary benefits in reducing cannabis use among AYAs with OUD.

This study has several important clinical implications for managing AYAs with OUD and co-occurring mental health and/or SUDs. The positive association between depression, PTSD, anxiety medication use, and longer retention highlights the importance of integrated care models that simultaneously address both mental health and opioid use disorders. Incorporating mental health care into OUD treatment settings may improve engagement, adherence, and overall treatment outcomes, particularly for those with comorbidities [43]. Medication-assisted treatment remains a cornerstone of OUD care. Integrating MOUD with evidence-based psychotherapies, such as cognitive behavioral therapy and motivational interviewing, can address the psychological aspects of substance use and foster behavioral change [44,45]. This requires building multidisciplinary teams that include medical, behavioral, and social service providers. These teams work together to address the medical, psychological, and social needs of patients. One key strategy could be the co-location of mental health and substance use treatment services within the same facility [46]. This physical proximity reduces logistical barriers, allowing patients to access comprehensive care without navigating separate systems. Data integration and sharing among healthcare providers also could contribute to coordinated care by minimizing duplication of services and improving communication [47]. Additionally, building community-based partnerships with social support organizations can extend care beyond the clinical setting [48]. The association between older age and improved medication adherence for OUD suggests that targeted interventions should focus on younger AYAs who may face more challenges with MOUD adherence. Tailored strategies, such as increased support from healthcare providers, peer groups, or digital health interventions, may help address adherence barriers. Moreover, taking MOUD significantly lowered the odds of positive UDT for THC, indicating the broader benefits of MOUD use beyond that for opioid dependence. Clinicians should consider the potential for MOUD to provide ancillary benefits when designing care plans for AYAs with OUD, especially those with polysubstance use.

This study is the first to comprehensively assess the association of mental health and substance use disorders, MOUD, and retention and UDT results among AYAs with OUD. By examining retention time and UDT results for MOUD as primary outcomes, we provided a holistic view of treatment engagement and adherence in this population. However, several limitations must be acknowledged. First, the retrospective design of this study cannot establish causal relationships between psychiatric comorbidities and patient outcomes. While we identified associations between mental health disorders and retention time, it remains unclear whether longer retention time was due to the presence of mental health comorbidities or the availability and quality of mental health services at the clinic. Unmeasured confounders, such as family support and socioeconomic status may have impacted retention time and treatment adherence as well. Future research should address these potential confounders. Another limitation of this study is the difficulty in interpreting the relationship between PTSD, alcohol use disorder, and longer retention. While our findings suggest an association between these disorders and increased retention time, it is plausible that this may indicate a greater need for mental health support rather than a direct reflection of treatment effectiveness. Additionally, the observed reduction in THC positivity associated with buprenorphine-naloxone use may serve as a proxy for overall treatment adherence rather than a direct pharmacological effect. Furthermore, we were unable to assess whether individuals with longer retention time received more intensive mental health care or had higher mental health service utilization, as these points of data were not documented in the electronic medical records used in this study. Consequently, it was not feasible to conduct additional analyses, such as stratifying retention by the intensity of mental health care or exploring dose–response relationships between medication adherence and retention outcomes. Future prospective studies are needed to investigate the potential mediating role of mental health care intensity and treatment adherence in the relationship between psychiatric comorbidities and treatment retention. Such studies should systematically capture data on mental health service utilization, medication adherence, and treatment intensity to better understand causal pathways. Additionally, the study was conducted at the SUD clinic within a single healthcare system, which may limit the generalizability of the findings to other settings. The sample may not fully represent the broader population of adolescents and young adults with OUD, particularly as the study population was predominantly White (94.9%), limiting applicability to more diverse racial and ethnic groups. Future studies should prioritize including diverse groups of AYAs to understand the varying factors influencing treatment engagement and adherence across different demographic groups or utilize comprehensive national datasets to improve generalizability. Future studies should also explore practical strategies for integrating mental health care and substance use treatment into OUD management for AYAs to improve treatment engagement and adherence.

## 4. Methods

This retrospective, longitudinal cohort study included AYAs aged 14 to 25 years who were enrolled in office-based opioid treatment (OBOT) at the Substance Use Treatment and Recovery (STAR) clinic from 1 January 2009, to 31 December 2022. A comprehensive description of the STAR clinic and its services was provided in previously published articles [49]. Patients were included if they had a confirmed diagnosis of OUD and complete medical records containing demographics, treatment engagement, comorbidities, and clinical assessments. Patients were excluded if they were not engaged in treatment at the STAR clinic for at least four weeks to ensure the inclusion of individuals with sufficient exposure to treatment, allowing for a meaningful evaluation of treatment outcomes. The study protocol was reviewed and approved by the Institutional Review Board (IRB) of The Research Institute at Nationwide Children’s Hospital. Ethical clearance was granted under the approval code STUDY00003322 on 1 June 2023.

### 4.1. Data Collection

Demographic data, including age, biological sex, race (White versus non-White), and insurance (no insurance, Medicaid/state children’s health insurance program (sCHIP), commercial, and dual Medicaid and commercial insurance) were extracted from the medical records. Clinical data collected for this study included appointment dates, UDT results, comorbidities, and medications. The presence of co-occurring mental health disorders and other SUDs was identified through the International Statistical Classification of Diseases and Related Health Problems, 9th and 10th revisions (ICD-9, ICD-10) codes. Mental health disorders included depression, anxiety, attention-deficit/hyperactivity disorder (ADHD), bipolar disorder, oppositional defiant disorder (ODD), and post-traumatic stress disorder (PTSD). SUDs include cannabis use disorder, alcohol use disorder, nicotine use disorder, and other substance use disorders (heroin, cocaine, benzodiazepines, methamphetamine/amphetamines, and kratom). These diagnoses were established at or prior to the time of enrollment in the clinic and thus were considered pre-existing comorbidities rather than conditions that developed during treatment. Selective Serotonin Reuptake Inhibitors (SSRIs), Serotonin and Norepinephrine Reuptake Inhibitors (SNRIs), benzodiazepines, and buspirone were commonly prescribed for anxiety-related conditions. SSRIs, SNRIs, and tricyclic antidepressants (TCAs) were frequently used to treat depression.

### 4.2. Outcomes

The primary outcomes were retention time in the clinic during the first treatment episode and the proportion of positive UDT results for MOUD, indicating adherence to MOUD. MOUD included buprenorphine-naloxone and naltrexone. The secondary outcomes included the proportion of positive UDT results for illicit substances and for tetrahydrocannabinol (THC) only. Retention time was defined as the period between the first and last clinic appointments during a patient’s first treatment episode, with an 8-week gap between appointments marking the end of the episode. Subsequent treatment episodes were excluded from analyses. UDT was performed at every clinic appointment by nurses. UDTs included screening patients for the use of MOUD, opioids (morphine, hydrocodone, oxycodone, heroin, etc.), THC, and stimulants (methamphetamine, cocaine, etc.), and the results were reported as positive or negative. Expected results were positive UDTs for MOUD and negative UDTs for illicit substances. Nonadherence was defined as having negative UDT results for MOUD. Additionally, positive UDT results for any illicit substance were classified as relapse. We did not specifically categorize relapses as episodic or sustained due to the retrospective nature of this study.

### 4.3. Statistical Analysis

All statistical analyses were performed using R version 4.1.0. Mean and standard deviation (SD), median and interquartile range (IQR) were used to summarize demographic and clinical characteristics as appropriate. Participants were divided into four groups based on the presence of comorbidities: (1) no comorbidities, (2) only mental health disorders, (3) only other SUDs, and (4) both mental health disorders and SUDs. Kruskal–Wallis tests were used to compare retention time, the proportion of positive UDT for MOUD, and the proportion of positive UDT for illicit substances across groups because the assumptions of normality and homogeneity of variance were not met. Multiple linear regression models and logistic regression models were used to identify factors significantly associated with retention time and positive UDT results for MOUD and illicit substances separately. Independent variables included demographics, comorbid diagnoses, medication used for mental health conditions, and types of MOUD. We used Bonferroni correction, with a *p*-value < 0.0125 considered statistically significant. 95% confidence intervals (CIs) were reported for coefficient.

## 5. Conclusions

Mental health and substance use disorders had significant impacts on retention time and adherence to MOUD. Depression, PTSD, and the taking medications for anxiety were associated with increased treatment retention. Additionally, older age and the use of medications for anxiety and depression were linked to higher adherence to MOUD. Conversely, cannabis use disorder was associated with shorter retention time. Furthermore, taking MOUD may reduce cannabis use. Future studies should investigate integrated care models and explore long-term treatment engagement strategies tailored to patients with co-occurring mental health and substance use disorders.

## Figures and Tables

**Table 1:** Demographic and clinical characteristics of adolescents and young adults with opioid use disorder.

	Overall (N = 157)	No Comorbidity (N = 26)	Patients with MHDs (N = 45)	Patients with SUDs (N = 37)	Patients with both Disorders (N = 49)	*p*
*Demographics*						
Age, years, mean (SD)	18.5 (1.9)	18.7 (1.9)	19.0 (1.8)	18.1 (1.5)	18.2 (2.2)	0.093
Sex, N (%)						0.974
Females	95 (60.5)	15 (57.7)	28 (62.2)	23 (62.2)	29 (59.2)	
Males	62 (39.5)	11 (42.3)	17 (37.8)	14 (37.8)	20 (40.8)	
Race, N (%)						0.282
White	149 (94.9)	26 (100.0)	44 (97.8)	34 (91.9)	45 (91.8)	
Non-White	8 (5.1)	0 (0.0)	1 (2.2)	3 (8.1)	4 (8.2)	
Insurance, N (%)						0.482
No insurance	4 (2.5)	0 (0.0)	0 (0.0)	1 (2.7)	3 (6.1)	
Medicaid/sCHIP	71 (45.2)	13 (50.0)	21 (46.7)	15 (40.5)	22 (44.9)	
Private/commercial	41 (26.1)	8 (30.8)	9 (20.0)	13 (35.1)	11 (22.4)	
Dual insurances	41 (26.1)	5 (19.2)	15 (33.3)	8 (21.6)	13 (26.5)	
*Mental Health Disorders*						
Number of MHDs mean (SD)	1.01 (1.10)	0 (0.0)	1.44 (0.59)	0 (0.0)	1.92 (1.11)	<0.001
Previous Suicidal Ideation, N (%)	7 (4.5)	0 (0.0)	1 (2.2)	0 (0.0)	6 (12.2)	0.015
ADHD/ADD, N (%)	23 (14.6)	0 (0.0)	9 (20.0)	0 (0.0)	14 (28.6)	<0.001
Taking ADHD medication, N (%)	13 (59.1)	0 (0.0)	6 (66.7)	0 (0.0)	7 (53.8)	0.023
Depression, N (%)	52 (33.1)	0 (0.0)	21 (46.7)	0 (0.0)	31 (63.3)	<0.001
Taking antidepressants, N (%)	42 (80.8)	0 (0.0)	18 (85.7)	0 (0.0)	24 (77.4)	<0.001
Anxiety, N (%)	60 (38.2)	0 (0.0)	28 (62.2)	0 (0.0)	32 (65.3)	<0.001
Taking anxiety medication, N (%)	47 (78.3)	0 (0.0)	23 (82.1)	0 (0.0)	24 (75.0)	<0.001
Bipolar disorder, N (%)	8 (5.1)	0 (0.0)	2 (4.4)	0 (0.0)	6 (12.2)	0.035
Taking bipolar medication, N (%)	7 (87.5)	0 (0.0)	2 (100.0)	0 (0.0)	5 (83.3)	0.081
PTSD, N (%)	7 (4.5)	0 (0.0)	4 (8.9)	0 (0.0)	3 (6.1)	0.149
ODD, N (%)	2 (1.3)	0 (0.0)	0 (0.0)	0 (0.0)	2 (4.1)	0.215
*Substance use disorder*						
Number of SUDs mean (SD)	0.65 (0.71)	0 (0.0)	0 (0.0)	1.14 (0.42)	1.22 (0.59)	<0.001
Nicotine use disorder, N (%)	12 (7.6)	0 (0.0)	0 (0.0)	4 (10.8)	8 (16.3)	0.009
Alcohol use disorder, N (%)	3 (1.9)	0 (0.0)	0 (0.0)	1 (2.7)	2 (4.1)	0.434
Cannabis use disorder, N (%)	19 (12.1)	0 (0.0)	0 (0.0)	4 (10.8)	15 (30.6)	<0.001
Other substance disorder, N (%)	68 (43.3)	0 (0.0)	0 (0.0)	33 (89.2)	35 (71.4)	<0.001
*MOUD Type*						*0.318*
No treatment, N (%)	2 (1.3)	0 (0.0)	0 (0.0)	0 (0.0)	2 (4.1)	
Buprenorphine-naloxone	139 (88.5)	22 (84.6)	41 (91.1)	36 (97.3)	40 (81.6)	
Naltrexone, N (%)	1 (0.6)	0 (0.0)	0 (0.0)	0 (0.0)	1 (2.0)	
Buprenorphine-naloxone and naltrexone, N (%)	15 (9.6)	4 (15.4)	4 (8.9)	1 (2.7)	6 (12.2)	

Abbreviations: MHDs: mental health disorders; SUDs: substance use disorders; ADHD: attention-deficit/hyperactivity disorder; ADD: attention deficit disorder; sCHIP: state children’s health insurance program; PTSD: post-traumatic stress disorder; ODD: oppositional defiant disorder; MOUD: medication for opioid use disorder; SD: standard deviation.

**Table 2:** Associations of demographic and clinical variables with retention time and proportion of positive urine drug tests for MOUD, illicit substances, and THC among adolescents and young adults with opioid use disorder.

	Retention Time		PPUDT for MOUD		PPUDT for Illicit Substances		PPUDT for THC	
Variables	Coefficient (95% CI)	*p*	Coefficient (95% CI)	*p*	Coefficient (95% CI)	*p*	Coefficient (95% CI)	*p*
Age	44.24 (−8.48, 96.97)	0.102	0.16 (0.02, 0.29)	0.023	−0.01 (−0.03, 0.02)	0.604	−0.01 (−0.04, 0.02)	0.596
Male	−157.33 (−364.09, 49.44)	0.138	0.32 (−0.22, 0.86)	0.254	0.07 (−0.03, 0.16)	0.154	0.14 (0.01, 0.27)	0.039
Non-white	−333.44 (−793.35, 126.48)	0.157	−0.12 (−1.31, 1.06)	0.838	0.19 (−0.01, 0.4)	0.068	0.25 (−0.05, 0.54)	0.101
Medicaid/sCHIP	267.29 (−385.69, 920.27)	0.424	0.27 (−1.4, 1.95)	0.748	0.22 (−0.07, 0.51)	0.139	0.37 (−0.03, 0.78)	0.075
Commercial insurance	325.42 (−340.18, 991.02)	0.339	0.82 (−0.88, 2.53)	0.346	0.06 (−0.23, 0.36)	0.667	0.15 (−0.27, 0.56)	0.493
Dual insurances	453.62 (−211.98, 1119.22)	0.184	0.28 (−1.43, 1.99)	0.749	0.17 (−0.12, 0.47)	0.258	0.29 (−0.12, 0.71)	0.169
Previous Suicidal Ideation	−189.22 (−681.53, 303.09)	0.452	−0.36 (−1.62, 0.9)	0.578	−0.2 (−0.42, 0.02)	0.076	0.08 (−0.24, 0.39)	0.626
ADHD/ADD	−53.11 (−340.87, 234.65)	0.718	−0.28 (−1.03, 0.47)	0.471	0.05 (−0.08, 0.18)	0.479	0.07 (−0.11, 0.25)	0.443
Taking medication for ADHD	171.62 (−287.09, 630.34)	0.472	0.11 (−0.15, 0.36)	0.420	−0.1 (−0.36, 0.16)	0.459	−0.13 (−0.5, 0.24)	0.501
Depression	220.33 (6.84, 433.82)	0.045	−0.19 (−0.75, 0.37)	0.498	−0.07 (−0.17, 0.03)	0.150	−0.04 (−0.17, 0.1)	0.611
Taking medication for depression	236.88 (−270.2, 743.95)	0.364	0.28 (0.09, 0.48)	0.006	−0.11 (−0.3, 0.09)	0.276	0.06 (−0.24, 0.36)	0.698
Anxiety	94.17 (−114.8, 303.13)	0.378	−0.22 (−0.76, 0.32)	0.427	0.02 (−0.07, 0.11)	0.684	0 (−0.13, 0.14)	0.966
Taking medication for anxiety	573.12 (179.09, 967.15)	0.006	0.4 (0.24, 0.57)	0.000	−0.14 (−0.33, 0.05)	0.162	0.11 (−0.15, 0.37)	0.428
PTSD	758.31 (279.77, 1236.86)	0.002	−0.14 (−1.4, 1.12)	0.829	−0.08 (−0.31, 0.14)	0.458	−0.03 (−0.34, 0.28)	0.846
Bipolar disorder	−211.21 (−672.91, 250.5)	0.371	−0.25 (−1.43, 0.94)	0.684	0.1 (−0.11, 0.31)	0.333	0.16 (−0.14, 0.45)	0.296
Taking medication for bipolar	225.86 (−1451.14, 1902.86)	0.801	0.77 (−0.02, 1.56)	0.103	0.2 (−0.6, 0.99)	0.648	−0.4 (−1.52, 0.72)	0.509
ODD	−495.38 (−1399.73, 408.97)	0.285	−0.33 (−2.66, 1.99)	0.779	−0.16 (−0.57, 0.25)	0.443	−0.33 (−0.91, 0.24)	0.257
Nicotine use disorder	157.56 (−224.78, 539.89)	0.421	−0.49 (−1.47, 0.49)	0.330	−0.03 (−0.2, 0.15)	0.759	−0.09 (−0.33, 0.15)	0.461
Alcohol use disorder	787.65 (54.52, 1520.78)	0.037	−0.2 (−2.11, 1.7)	0.835	0.19 (−0.15, 0.52)	0.275	0 (−0.47, 0.48)	0.988
Cannabis use disorder	−357.49 (−664.49, −50.5)	0.024	−0.53 (−1.36, 0.31)	0.218	0.04 (−0.1, 0.18)	0.591	0.23 (0.03, 0.42)	0.023
Other substance disorder	−78.26 (−283.33, 126.81)	0.456	0.26 (−0.27, 0.79)	0.336	−0.01 (−0.1, 0.08)	0.797	−0.1 (−0.23, 0.03)	0.124
Buprenorphine-naloxone	411.1 (−499.62, 1321.82)	0.378	−0.94 (−4.22, 2.35)	0.576	0.13 (−0.28, 0.53)	0.537	−0.69 (−1.26, −0.12)	0.019
Buprenorphine-naloxone and naltrexone	1 (−1565.18, 1567.18)	0.999	−0.26 (−1.18, 0.65)	0.574	0.07 (−0.63, 0.77)	0.844	−0.62 (−1.22, −0.02)	0.042

Abbreviations: CI: confidence interval; ADHD: attention-deficit/hyperactivity disorder; ADD: attention deficit disorder; ODD: oppositional defiant disorder; PTSD: post-traumatic stress disorder; sCHIP: state children’s health insurance program; PPUDT: proportion of positive urine drug tests; MOUD: medication for opioid use disorder; THC: tetrahydrocannabinol.

## Data Availability

The data presented in this study are available on request from the corresponding author due to privacy, legal and ethical reasons.

## Abbreviations

The following abbreviations are used in this manuscript:

AYA	Adolescents and Young Adults
OUD	Opioid Use Disorder
SUD	Substance Use Disorder
PTSD	Post-Traumatic Stress Disorder
UDT	Urine Drug Test
MOUD	Medication for Opioid Use Disorder
STAR	Substance Use Treatment and Recovery (Clinic)
OBOT	Office-Based Opioid Treatment
sCHIP	State Children’s Health Insurance Program
ICD-9	International Classification of Diseases, 9th Revision
ICD-10	International Classification of Diseases, 10th Revision
ADHD	Attention-Deficit/Hyperactivity Disorder
ODD	Oppositional Defiant Disorder
SSRIs	Selective Serotonin Reuptake Inhibitors
SNRIs	Serotonin and Norepinephrine Reuptake Inhibitors
TCAs	Tricyclic Antidepressants
THC	Tetrahydrocannabinol
IQR	Interquartile Range
CI	Confidence Interval
SD	Standard Deviation
